# Medikamentöse Sekundärprävention bei Patienten mit peripherer arterieller Verschlusskrankheit: Eine sekundärdatenbasierte Analyse

**DOI:** 10.1007/s00059-020-04998-w

**Published:** 2020-11-18

**Authors:** Katrin Gebauer, Kerstin Wintersohl, Rike Kraska, Katja Kortendick, Ulrike Fahrland, Eva Freisinger, Matthias Meyborg, Jacqueline Stella, Christiane Engelbertz, Holger Reinecke, Nasser Malyar

**Affiliations:** 1 Klinik für Kardiologie I: Koronare Herzkrankheit, Herzinsuffizienz und Angiologie, Universitätsklinikum Münster, Albert-Schweitzer-Campus 1, Geb. A1, 48149 Münster, Deutschland; 2 Verordnungsmanagement, Kassenärztliche Vereinigung Westfalen-Lippe, Dortmund, Deutschland

**Keywords:** Atherosklerose, Risikofaktoren, Versorgungsforschung, Arzneiverordnungen, Prognose, Atherosclerosis, Risk factors, Health services research, Drug prescriptions, Prognosis

## Abstract

**Hintergrund:**

Die periphere arterielle Verschlusskrankheit (pAVK) ist eine atherosklerotische Gefäßerkrankung mit hoher Morbidität und Mortalität. Eine konsequente medikamentöse Sekundärprävention gehört zur essenziellen und evidenzbasierten Therapie der pAVK. Das Ziel der vorliegenden Studie war es, den Status quo der medikamentösen Sekundärprävention anhand von Rezepteinlösungen zu ermitteln.

**Methoden:**

Basierend auf Sekundärdaten der Kassenärztlichen Vereinigung Westfalen-Lippe (KVWL), wurden im Zeitraum von 2014 bis 2017 Patienten mit einer gesicherten pAVK-Kodierung (I70.2-/I73.9-) identifiziert und deren Rezepteinlösung bezüglich Thrombozytenaggregationshemmern (TAH), oralen Antikoagulanzien, lipidmodifizierender Medikation (LLT) sowie ACE(„angiotensin-converting enzyme“)-Hemmer im 4. Quartal nach der Diagnosekodierung erfasst.

**Ergebnisse:**

Im Diagnosezeitraum 2014/2015 hatten im Einzugsgebiet KVWL 238.397 Patienten eine pAVK. Der Anteil an eingelösten Rezepten betrug im 4. Quartal nach der Diagnosestellung 25,9 % für LLT, 13,6 % für Acetylsalicylsäure, 4,5 % für Clopidogrel, 5,5 % für Vitamin-K-Antagonisten (VKA), 3,5 % für nicht-Vitamin-K-abhängige orale Antikoagulanzien (NOAK) und 26,8 % für ACE-Hemmer. Im Verlauf von 3 Jahren (*n* = 241.375 Patienten mit pAVK 2016/2017) stieg der Anteil an eingelösten Rezepten bis auf VKA für alle anderen Substanzen an (*p* < 0,001), wobei der größte relative Anstieg bei NOAK zu verzeichnen war (relativer Anstieg um 81,7 %).

**Schlussfolgerung:**

Die leitliniengerechte medikamentöse Sekundärprävention bei pAVK-Patienten in Deutschland ist weiterhin verbesserungswürdig. Eine konsequente Umsetzung evidenzbasierter medikamentöser Sekundärprävention beherbergt ein großes Potenzial zur Verbesserung der Gesamtprognose der pAVK-Patienten.

## Abstract (en)

**Background:**

Peripheral arterial occlusive disease (PAOD) is an atherosclerotic vascular disease with high morbidity and mortality. A consistent medication-based secondary prevention is part of the essential and evidence-based treatment of PAOD. The aim of this study was to ascertain the status quo of medicinal secondary prevention based on submitted prescriptions.

**Methods:**

In the time period from 2014 to 2017 patients with a confirmed PAOD coding (I70.2-/I73.9-) were identified based on secondary data of the Association of Statutory Health Insurance Physicians Westphalia-Lippe (KVWL). The prescriptions submitted with respect to platelet inhibitors, oral anticoagulants, lipid lowering therapy (LLT) and angiotensin-converting enzyme (ACE) inhibitors in the fourth quarter year after diagnosis coding were collated.

**Results:**

In the diagnosis period 2014/2015 a total of 238,397 patients had PAOD in the catchment area of the KVWL. The proportion of submitted prescriptions in the fourth quarter year after diagnosis was 25.9% for LLT, 13.6% for acetylsalicylic acid, 4.5% for clopidogrel, 5.5% for vitamin K antagonists (VKA), 3.5% for non-vitamin K‑dependent oral anticoagulants (NOAC) and 26.8% for ACE inhibitors. Over the course of the 3 years (*n* = 241,375 patients with PAOD 2016/2017) the proportion of submitted prescriptions for all substances except VKA increased (*p* < 0.001), whereby the largest relative increase was noted for NOAC (relative increase of 81.7%).

**Conclusion:**

The guideline-conform medicinal secondary prevention in patients with PAOD in Germany is still in need of improvement. A consistent implementation of evidence-based medicinal secondary prevention harbors a great potential for improvement of the overall prognosis in patients with PAOD.

## Einleitung

Die periphere arterielle Verschlusskrankheit (pAVK) ist eine mit dem Alter an Häufigkeit zunehmende atherosklerotische Gefäßerkrankung, die unabhängig von konventionellen Risikofaktoren und anderen Komorbiditäten mit erhöhter kardiovaskulärer Morbidität und Mortalität einhergeht [1]. Eine leitliniengerechte Sekundärprophylaxe ist eine essenzielle und effektive Strategie, um bei Patienten mit manifester Atherosklerose kardiovaskuläre Ereignisse wie Herzinfarkte und Schlaganfälle, aber auch negative Auswirkungen der pAVK wie ischämische Extremitätenamputationen und wiederholte Revaskularisationsprozeduren zu verhindern [2–6]. Zur leitliniengerechten Sekundärprävention zählt neben der Lebensstilmodifikation mit Ernährungsoptimierung und regelmäßigem Gehtraining eine strikte Nikotinkarenz, eine optimale pharmakologische Einstellung kardiovaskulärer Risikofaktoren, insbesondere des Diabetes mellitus, der arteriellen Hypertonie und der Dyslipidämie. Der protektive Einfluss von Thrombozytenaggregationshemmern (TAH), lipidmodifizierenden Substanzen wie den 3‑Hydroxy-3-Methylglutaryl-Coenzym-A-Reduktase-Inhibitoren (Statinen) und ACE(„angiotensin-converting enzyme“)-Hemmern ist in zahlreichen Studien gut belegt [2, 3, 7].

Die überwältigende Evidenz des protektiven Effekts dieser Substanzen sowie deren Klasse-1A-Empfehlung in den entsprechenden Leitlinien (S3-Leitlinie pAVK, ESC[European Society of Cardiology]-Leitlinie, AHA[American Heart Association]/ACC[American College of Cardiology]-Leitlinie [8–10]) stehen im Widerspruch zur unzureichenden Implementierung der medikamentösen Sekundärprävention im klinischen Alltag [11, 12].

Das Ziel der vorliegenden Arbeit war es, den aktuellen Stand und den Trend der evidenzbasierten medikamentösen Sekundärprävention bei pAVK-Patienten anhand von eingelösten Medikationsverordnungen im Einzugsbereich der Kassenärztlichen Vereinigung Westfalen-Lippe (KVWL) zu ermitteln.

## Methode

Datengrundlage der Analysen waren pseudonymisierte Arzneiverordnungsdaten (AVD) gemäß § 300 Abs. 2 SGB V und pseudonymisierte ambulante Versorgungsdaten (VDA) gemäß §295 SGB V der KVWL der Jahre 2014 bis 2018.

Die Daten von Arzneimittelrezepten, die von gesetzlich Versicherten in Deutschland in Apotheken eingelöst werden, werden für die Abrechnung der Apotheken mit der gesetzlichen Krankenversicherung (GKV) von Apothekenrechenzentren elektronisch erfasst und darüber hinaus den Kassenärztlichen Vereinigungen (KVen) gemäß § 300 SGB V zur Verfügung gestellt. Als zentrale Stelle zur Datenaufbereitung haben die KVen das Zentralinstitut für die kassenärztliche Versorgung in Deutschland (Zi) beauftragt. Hierfür werden dem Zi neben den AVD die vertragsärztlichen Abrechnungsdiagnosen der ambulanten VDA gemäß §295 SGB V von den KVen zur Verfügung gestellt. Über eine sog. Vertrauensstelle des Zi werden die Daten nach den datenschutzrechtlichen Vorgaben zusammengeführt, der Versichertenbezug wird vollständig pseudonymisiert, und die zusammengefügten Datensätze werden anschließend an die KVen übermittelt.

**Figure 1:**
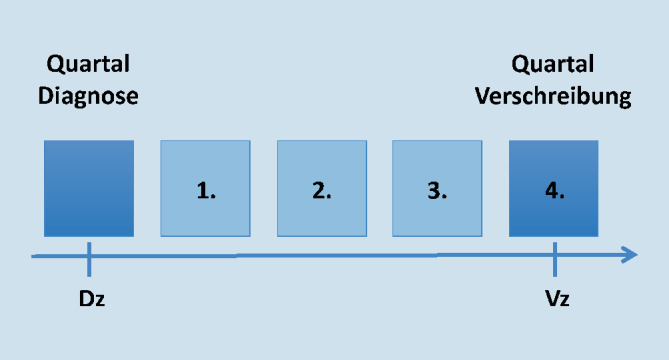

Alle Patienten im Einzugsgebiet der KVWL, die in den Jahren 2014 bis 2017 mit mindestens 1‑maliger gesicherter Kodierung des ICD(International Statistical Classification of Diseases and Related Health Problems)-10-Codes I70.2- (Atherosklerose der Extremitätenarterien) oder I73.9- (periphere Gefäßkrankheit) identifiziert wurden, bildeten die Zielkohorte. Für jeden Patienten mit der Diagnose einer pAVK wurden die eingelösten Verordnungen für lipidmodifizierende Medikation (Statine, Ezetimib, PCSK9[Proproteinkonvertase Subtilisin/Kexin Typ 9]-Inhibitoren, ACE-Hemmer, TAH [Acetylsalicylsäure, ASS], Clopidogrel) und orale Antikoagulanzien (Vitamin-K-Antagonisten [VKA], Dabigatran, Rivaroxaban, Apixaban, Edoxaban) ermittelt. Um den Effekt einer Tendenz zur höheren Adhärenz kurz nach einer Diagnosestellung zu minimieren, wurden die entsprechenden Rezepteinlösungen im 4. Quartal nach der Diagnosestellung für die Analyse herangezogen (Abb. 1).

### Statistische Analyse

Neben einer deskriptiven Betrachtung wurden Unterschiede zwischen den Geschlechtern und Zeiträumen mit Hilfe des χ^2^-Tests (Signifikanzniveau: *p* ≤ 0,05) untersucht.

## Ergebnisse

Die Gesamtzahl der Patienten mit einer ICD-10-Codierung I70.2-/I73.9- lag im Diagnosezeitraum 2014/15 bei 238.397 und stieg im Diagnosezeitraum 2016/17 auf 241.375 an. Der Anteil der pAVK-Patienten an der Gesamtzahl aller Patienten mit einer gesicherten Diagnose im Einzugsgebiet der KVWL stieg innerhalb desselben Zeitraums von 2,38 auf 2,65 % an.

**Figure 2:**
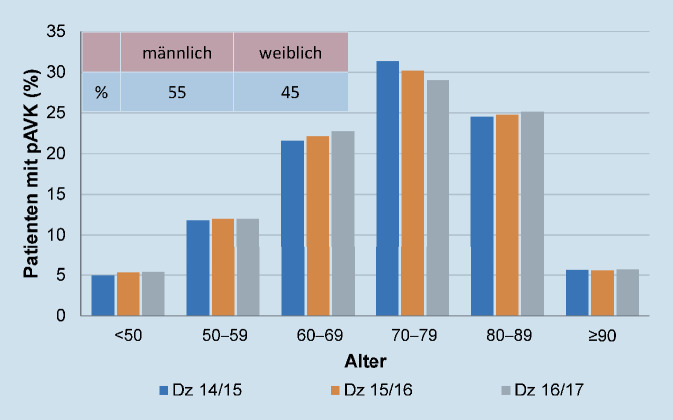

Die Altersverteilung der Kohorte in Lebensdekaden ist in Abb. 2 dargestellt. In der Dekade von 70 bis 79 Jahren zeigt sich ein deutlicher Altersgipfel, gefolgt von den Dekaden der 80- bis 89-Jährigen und der 60- bis 69-Jährigen. Hinsichtlich der Geschlechtsverteilung lag über dem gesamten Beobachtungszeitraum eine konstante Relation von 55 % männlichen zu 45 % weiblichen Patienten vor.

**Figure 3:**
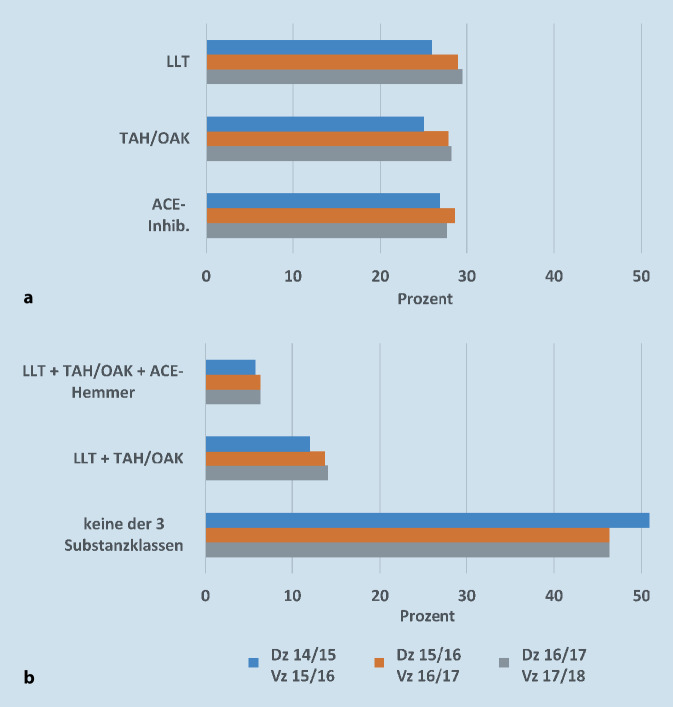

Die Anzahl eingelöster Verordnungen und deren prozentuale Häufigkeit innerhalb der Gesamtkohorte für die einzelnen Medikamente in den Jahreszeiträumen 2015/16 bis 2017/18 sind in Tab. 1 dargestellt. Lediglich 29,5 % aller Patienten des Diagnosezeitraums 2016/17 lösten 4 Quartale später ein Rezept für lipidmodifizierende Medikation, 14,5 % für ASS, 4,7 % für Clopidogrel, 11,3 % für Antikoagulanzien und 27,7 % für ACE-Hemmer ein. Nur 28,2 % der Patienten lösten eine Verordnung für Antikoagulanzien und/oder TAH ein. Der Anteil derjenigen Patienten, die eine Verordnung für alle 3 in den Leitlinien empfohlenen Substanzklassen (lipidmodifizierende Medikation, TAH/Antikoagulanzien, ACE-Hemmer) einlösten, betrug 6,4 %, wohingegen der Anteil derjenigen, die für keine der 3 oben genannten Substanzklassen ein Rezept einlösten, bei 46,4 % lag (Tab. 1; Abb. 3).

**Table 1:** 

	Dz: 2014/15 Vz: 2015/16	Dz: 2015/16 Vz: 2016/17	Dz: 2016/17 Vz: 2017/18	*p*-Wert
Patienten	238.397	237.499	241.375	–
Statin, *n* (%)	61.188 (25,67)	68.140 (28,69)	70.369 (29,15)	<0,001
Ezetimib, *n* (%)	1.191 (0,50)	1.461 (0,62)	1.898 (0,79)	<0,001
Statin u/o Ezetimib, *n* (%)	61.729 (25,89)	68.781 (28,96)	71.117 (29,46)	<0,001
LLT, *n* (%)	61.730 (25,89)	68.796 (28,97)	71.153 (29,48)	<0,001
ASS, *n* (%)	32.389 (13,59)	34.907 (14,70)	34.929 (14,47)	<0,001
Clopidogrel, *n* (%)	10.621 (4,46)	11.463 (4,83)	11.393 (4,72)	<0,001
TAH, *n* (%)	40.498 (16,99)	43.666 (18,39)	43.668 (18,09)	<0,001
VKA, *n* (%)	13.159 (5,52)	13.492 (5,68)	12.388 (5,13)	<0,001
NOAK, *n* (%)	8.227 (3,45)	11.908 (5,01)	15.133 (6,27)	<0,001
VKA u/o NOAK, *n* (%)	21.233 (8,91)	25.217 (10,62)	27.322 (11,32)	<0,001
TAH u/o Antikoagulation, *n* (%)	59.559 (24,98)	66.203 (27,88)	68.093 (28,21)	<0,001
LLT + (TAH u/o Antikoagulation), *n* (%)	28.638 (12,01)	32.573 (13,72)	33.925 (14,05)	<0,001
ACE-Hemmer, *n* (%)	63.883 (26,80)	68.039 (28,65)	66.875 (27,71)	<0,001
LLT + (TAH u/o Antikoagulation) + ACE-Hemmer, *n* (%)	13.771 (5,78)	15.123 (6,37)	15.355 (6,36)	<0,001
Keine der oben genannten Substanzklassen, *n* (%)	121.480 (50,96)	109.985 (46,31)	111.869 (46,35)	<0,001

*ACE* „angiotensin-converting enzyme“, *ASS* Acetylsalicylsäure, *LLT* „lipid-lowering therapy“ (Statin und/oder Ezetimib und/oder PCSK9[Proproteinkonvertase Subtilisin/Kexin Typ 9]-Inhibitor), *NOAK* nicht-Vitamin-K-abhängige orale Antikoagulanzien, *TAH* Thrombozytenaggregationhemmer (ASS und/oder Clopidogrel), *VKA* Vitamin-K-Antagonisten, *Antikoagulation* VKA und/oder NOAK, *u/o* und/oder

Es zeigte sich im Verlauf von 3 Jahren bei allen Substanzklassen ein signifikanter Anstieg (*p* < 0,001). Lediglich bei VKA gab es einen signifikanten Rückgang von 5,52 auf 5,13 % (*p* < 0,001; Tab. 1). Hingegen zeigte sich bei den nicht-Vitamin-K-abhängigen oralen Antikoagulanzien (NOAK) der größte relative Anstieg von 3,45 auf 6,27 % (+81,7 %).

## Diskussion

Die vorliegenden Ergebnisse verdeutlichen die immer noch dringend optimierungsbedürftige sekundärpräventive Versorgungslage von Patienten mit pAVK im realen klinischen Alltag: Demnach erhielten lediglich 29,2 % dieser Patientengruppe (Diagnosezeitraum: 2016/17, Verordnungszeitraum: 2017/18) Statine, 18,1 % einen TAH (ASS und/oder Clopidogrel) sowie 11,3 % eine orale Antikoagulation mit VKA und/oder einem der NOAK. Eine Kombination aus einer lipidmodifizierenden Medikation und Antikoagulation/TAH konnte nur bei 14,1 % der Patienten verzeichnet werden. Über alle Substanzklassen betrachtet erhielt nur gut jeder zweite pAVK-Patient (54 %) mindestens eines der leitliniengerechten Medikamente. Dies stellt wahrscheinlich eine Ursache dar, warum die Prognose von Patienten mit pAVK mit Einjahressterblichkeitsraten je nach Stadium bis zu 40 % und Einjahresamputationsraten bis zu 60 % sehr schlecht ist [13].

Unsere Studienergebnisse zeigen, dass die tatsächliche, im Alltag praktizierte medikamentöse Sekundärprävention bei Patienten mit pAVK noch geringer zu sein scheint, als bisher postuliert. Die aus prospektiven klinischen Studien und Registern gewonnenen Daten repräsentieren eine Selektion und sind wahrscheinlich insofern einem Bias unterworfen, als die an den Studien teilnehmenden Zentren allein schon studienbedingt eine konsequentere Sekundärprävention und eine forciertere Behandlungsintensität verfolgen, als dies im klinischen Alltag der Fall ist.

Zwar stiegen die Verordnungen im Beobachtungszeitraum moderat an, der Anteil derjenigen mit einer leitliniengerechten, medikamentösen Sekundärprävention ist jedoch weiterhin weit entfernt von einem optimalen Wert, selbst wenn man die dieser Studie zugrunde liegenden Limitationen (siehe Abschnitt „Limitationen“) berücksichtigt.

Diese Diskrepanz zwischen den evidenzbasierten und leitliniengestützten Empfehlungen und der im klinischen Alltag praktizierten Realität scheint ein über die letzten 3 Dekaden persistierendes, über die Landesgrenzen und Gesundheitssysteme hinausgehendes Phänomen zu sein. Eine Unterversorgung der pAVK-Patienten hinsichtlich der Sekundärprävention ist bereits früher, nicht nur in Deutschland, sondern auch in anderen Ländern und Gesundheitssystemen, beschrieben worden. Die getABI-Studie [11] hat gezeigt, dass nur jeder zweite pAVK-Patient in Deutschland einen TAH erhielt. Noch schlechter war die Einnahmerate für Statine mit nur 23 %. Bemerkenswert war die Tatsache, dass pAVK-Patienten im Vergleich zu Patienten mit koronarer Herzkrankheit (KHK) und zerebrovaskulärer Verschlusskrankheit (cAVK) am schlechtesten versorgt waren. In einer weiteren, bundesweiten und ebenfalls auf Rezeptverordnungen basierten Analyse erhielten nur 34,8 % der 3352 pAVK-Patienten ein Statin [12]. Selbst in Ländern mit traditionell guter medizinischer Versorgungslage wie in Dänemark ist die Sekundärprävention bei pAVK mangelhaft. Gasse et al. [14] konnten in einer auf Rezeptverordnungen basierten Studie in Dänemark zeigen, dass 6 Monate nach der Erstdiagnose einer pAVK nur noch 26 % der Patienten einen TAH einnahmen und die Statineinnahmerate bei lediglich 10 % lag. Ähnlich wie in der getABI-Studie in Deutschland hatten Patienten mit einem stattgehabten Myokardinfarkt eine wesentlich bessere, wenn auch nicht optimale Quote mit 55 % für TAH bzw. 46 % für Statine. In einer weiteren, landesweiten dänischen Kohortenstudie (*n* = 197.913) erhielten 36,7 % der 34.164 pAVK-Patienten einen TAH, 23,9 % ein Statinpräparat. Auch in dieser Studie war die Quote der Einnahme bei pAVK-Patienten schlechter als bei KHK-Patienten (44 % bzw. 26,3 %, *p* < 0,001; [15]). In einer aktuellen Analyse an 70.753 pAVK-Patienten aus Großbritannien zeigte sich, dass im Langzeitverlauf die Statin- und TAH-Verschreibungs-Rate im Jahre 2006 bei 45 % bzw. 38,2 % lag [16]. Eine ähnlich unzureichende Sekundärprävention bei pAVK-Patienten ist auch für die Vereinigten Staaten beschrieben worden [17, 18]. Die Tatsache, dass von den Atherothrombosepatienten diejenigen mit einer pAVK die niedrigste medikamentöse Sekundärpräventionsrate aufweisen, legt die Vermutung nahe, dass die pAVK weiterhin als das „Stiefkind“ der Atherothrombose erachtet und entsprechend behandelt wird [19], obwohl hinreichend bekannt ist, dass das kardiovaskuläre Risiko gerade bei pAVK-Patienten am höchsten ist [15, 17, 18].

Gleichwohl offenbart die aktuelle unzureichende Versorgungssituation jedoch auch eine Chance und zeigt das Potenzial, das es durch eine konsequentere Umsetzung der evidenzbasierten Empfehlungen auszuschöpfen gilt. Eine verbesserte und suffiziente Sekundärprävention ist nicht nur aus der Patientenperspektive wichtig, indem sie eine Reduktion der kardiovaskulären Ereignisse, eine Verbesserung der Gehstrecke, eine Reduktion der Amputationsrate und eine Verbesserung der Lebensqualität bewirkt. Sie führt auch zu einer Entlastung der Kostenträger, da Folge- und Rezidivereignisse wie Herzinfarkte, Schlaganfälle, Amputationen, wiederholte stationäre Aufenthalte und Revaskularisationen samt ihrer Folgekosten reduziert werden. Hier sind insbesondere eine zeitnahe Diagnosestellung und die Einleitung einer leitliniengerechten Sekundärprävention wichtig, da der Effekt einer Risikoreduktion desto größer ist, je früher mit den Präventionsmaßnahmen begonnen wird.

Die Gründe für die Diskrepanz zwischen Leitlinienempfehlung und tatsächlicher Versorgungslage bei der medikamentösen Sekundärprophylaxe bei pAVK-Patienten sind vielschichtig. Das Bewusstsein für die Notwendigkeit einer erforderlichen Sekundärprävention bei einer pAVK auf Arzt- wie auch auf Patientenseite scheint nicht so weit verbreitet zu sein wie bei der KHK, obwohl die empfohlenen Medikationsklassen überwiegend deckungsgleich sind (TAH, Statine, ACE-Hemmer). Die mediale Präsenz der KHK durch Stiftungen, Selbsthilfegruppen und durch die Kostenträger unterstützte Disease-Management-Programme haben die Akzeptanz der Therapie einer KHK inklusive der Sekundärprävention sowohl in der Ärzteschaft als auch bei den Patienten erheblich erhöht. Patienten mit pAVK sind häufig alt, multimorbide und polypharmaziebedürftig. Das alles sind Faktoren, die nachweislich mit einer reduzierten Compliance assoziiert sind [20–22].

Ansätze zur Verbesserung der Sekundärprävention bei pAVK-Patienten sind ähnlich vielschichtig wie die zugrunde liegenden Ursachen. Neben dem Schärfen des Bewusstseins für die Bedeutung der pAVK – auf Patienten- wie auf Ärzteseite – könnte eine Anbindung dieser Hochrisikopatienten an ein Disease-Management-Programm pAVK eine Verbesserung darstellen. Eine erfolgsabhängige Vergütung (im Sinne von „pay for performance“) wie z. B. in Dänemark [15] wäre auch eine Möglichkeit, die leitliniengerechte Therapie im Praxisalltag flächendeckend zu etablieren.

Nicht zuletzt haben sich Kooperationsmodelle zwischen Ärzten und Apothekern zur Medikationsadhärenz bei chronischen kardiovaskulären Erkrankungen als vorteilhaft erwiesen, sodass dieser Baustein sich ebenfalls als Teil einer multimodalen Strategie zur Verbesserung der Behandlungsqualität von Gefäßpatienten erweisen könnte [23].

## Limitationen

Unsere Studie weist einige Limitationen auf, die bei der Beurteilung der vorliegenden Ergebnisse Berücksichtigung finden müssen: Die landesgrenzen- bzw. KV-übergreifende Versorgung von Patienten (z. B. Diagnosestellung in einem und Rezepteinlösung in einem anderen KV-Bereich) kann zu einer Verzerrung der Daten im Sinne einer scheinbar geringeren Sekundärprävention führen. ASS als apothekenpflichtiges, nicht aber verschreibungspflichtiges Arzneimittel (Patienten könnten ASS ohne Rezept erwerben) sowie die Betrachtung der Rezepteinlösung ausschließlich im 4. Quartal nach Diagnosestellung (bei großen Arzneimittelpackungen oder bei 2 zeitlich nahe beieinanderliegenden Rezeptintervallen ist nicht zwingend ein Rezept in jedem Quartal nötig) können ebenfalls die geringen Quoten insbesondere der ASS-/TAH- und Antikoagulanzien-Rezept-Einlösung mitbedingt haben, wobei sich nach Heranziehen der Rezepteinlösungsdaten des 5. Quartals nach Diagnosestellung die Häufigkeit um etwa 8 % erhöhte und somit die Ergebnisse immer noch weit vom Erreichen einer Leitlinienadhärenz entfernt sind. Wir können zwar den Effekt der Verzerrung durch die oben erwähnten Faktoren auf die Studienergebnisse nicht quantifizieren, es ist jedoch davon auszugehen, dass diese Faktoren die De-facto-Versorgungssituation eher falsch-niedrig erscheinen lassen. Auf der anderen Seite ist es durchaus möglich, dass die eingelösten Verordnungen nicht primär in der Diagnose einer Gefäßerkrankung mit den ICD I70.2- und I73.9- begründet liegen. So sind Komorbiditäten wie arterielle Hypertonie, KHK oder cAVK ebenso mögliche Gründe für die Verordnung der hier untersuchten Substanzklassen. In dem Fall wäre die Quote derjenigen, die aufgrund ihrer pAVK eine leitliniengerechte medikamentöse Sekundärprophylaxe erhielten, eigentlich geringer, als angegeben.

Wir können keine verlässlichen Aussagen zur Adhärenz der Patienten ableiten. So kann weder eruiert werden, in welcher Häufigkeit zwar eine Rezeptierung einer Medikation erfolgte, der Patient diese aber nicht in der Apotheke eingelöst hat, noch wie viele Patienten zwar ein Rezept einlösten, die Medikamente aber gar nicht oder nur unregelmäßig eingenommen haben. Ob das Problem der unzureichenden Sekundärprävention schließlich auf der Seite der Ärzte oder der Patienten oder in unterschiedlicher Gewichtung sogar durch beide begründet ist, lässt sich aus den vorliegenden Daten nicht ableiten. Darüber hinaus liegen trotz der großen Fallzahl ausschließlich Daten aus dem Einzugsbereich der KVWL vor.

Ein weiterer Einflussfaktor liegt möglicherweise in der Art der Atherosklerosemanifestation begründet. Während es für die KHK breite Öffentlichkeitskampagnen, Stiftungsveranstaltungen und Selbsthilfegruppenaktivitäten gibt, sind für die pAVK derartige Maßnahmen zur Bewusstseinsschärfung und Unterstützung bei der Krankheitsbewältigung Mangelware. So kann eine KHK in der Gesamtwahrnehmung der Patienten mit allen zur Verfügung stehenden Therapiemöglichkeiten und supportiven Angeboten positiv konnotiert sein, wohingegen die pAVK mit den schweren Manifestationen inklusive „Raucherbein“ und der schlechten Prognose negativ besetzt ist, was zu einer niedrigeren Therapietreue führen kann.

## Fazit für die Praxis

- Hinsichtlich der medikamentösen Sekundärprävention bei peripherer arterieller Verschlusskrankheit (pAVK) besteht weiterhin eine große Diskrepanz zwischen den evidenzbasierten Leitlinienempfehlungen und deren konsequenter Umsetzung im klinischen Alltag.
- Eine auf die Ursachen fokussierte Forschung ist notwendig, um die Gründe für die unzureichende Umsetzung der evidenzbasierten Empfehlungen zu identifizieren.
- In einem Folgeschritt wären dann Strategien zu entwickeln, diese Ursachen anzugehen, um das volle protektive Potenzial einer leitliniengerechten medikamentösen Sekundärprävention auszuschöpfen und damit die Gesamtprognose der pAVK-Patienten hinsichtlich kardiovaskulärer und extremitätenbezogener Ereignisse zu verbessern.
